# A qualitative study on the adaptation of community programmes for the promotion of early detection and health-seeking of perinatal depression in Nepal

**DOI:** 10.1186/s12905-024-03122-y

**Published:** 2024-05-04

**Authors:** Prasansa Subba, Petal Petersen Williams, Nagendra Prasad Luitel, Mark J. D. Jordans, Erica Breuer

**Affiliations:** 1 Research Department, Transcultural Psychosocial Organization Nepal, Kathmandu, Nepal; 2 Department of Primary Care and Mental Health, Institute of Population Health, University of Liverpool, Liverpool, United Kingdom; 3 Mental Health, Alcohol, Substance Use and Tobacco Research Unit, South African Medical Research Council, Cape Town, South Africa; 4 Department of Global Health, Institute for Life Course Health Research, Stellenbosch University, Cape Town, South Africa; 5 Centre for Global Mental Health, King’s College London, London, United Kingdom; 6 Department of Medicine and Public Health, College of Health, Medicine and Wellbeing, The University of Newcastle, Callaghan, Australia

**Keywords:** Perinatal depression, Detection, Awareness, Health seeking, Community mental health, Task sharing, Nepal

## Abstract

**Background:**

Despite the high burden of perinatal depression in Nepal, the detection rate is low. Community-based strategies such as sensitization programmes and the Community Informant Detection Tool (CIDT) have been found to be effective in raising awareness and thus promoting the identification of mental health problems. This study aims to adapt these community strategies for perinatal depression in the Nepalese context.

**Methods:**

We followed a four-step process to adapt the existing community sensitization program manual and CIDT. *Step 1* included in-depth interviews with women identified with perinatal depression (*n*=36), and focus group discussions were conducted with health workers trained in community mental health (*n*=13), female community health volunteers (FCHVs), cadre of Nepal government for the prevention and promotion of community maternal and child health (*n*=16), and psychosocial counsellors (*n*=5). We explored idioms and understanding of depression, perceived causes, and possible intervention. *Step 2* included draft preparation based on the qualitative study. *Step 3* included a one-day workshop with the psychosocial counsellors (*n*=2) and health workers (*n*=12) to assess the understandability and comprehensiveness of the draft and to refine the content. A review of the CIDT and community sensitization program manual by a psychiatrist was performed in *Step 4.*

**Results:**

The first step led to the content development for the CIDT and community sensitization manual. Multiple stakeholders and experts reviewed and refined the content from the second to fourth steps. Idioms of depression and commonly cited risk factors were incorporated in the CIDT. Additionally, myths of perinatal depression and the importance of the role of family were added to the community sensitization manual.

**Conclusion:**

Both the CIDT and community sensitization manual are grounded in the local context and are simple, clear, and easy to understand.

**Supplementary Information:**

The online version contains supplementary material available at 10.1186/s12905-024-03122-y.

## Background

Women are more at risk of depression and anxiety than men [1, 2], and the risk is even higher during the perinatal period – defined as the period starting from pregnancy until a year after the delivery of a child [3–6]. Globally, approximately 12% of women suffer from perinatal depression [7], but the prevalence is higher in low- and middle-income countries (LMICs) than in high-income countries [7, 8]. A recent systematic review estimated that one-fourth of women in LMICs experience perinatal depression [8], and it is the leading cause of disability among perinatal women [9]. Despite the high burden, detection and treatment of perinatal depression is less common in LMICs [10, 11]. Untreated perinatal depression is marked by higher levels of disability in mothers, impaired growth of their children, and disrupted relationships with family members [12–15].

Evidence suggests that there is a large "treatment gap" (difference between those needing care and those receiving it) in mental health. The World Health Organization (WHO) introduced the mental health Gap Action Programme (mhGAP), which advocated for scaling basic mental health services at the community level by training and supporting primary-level health workers [16]. A research project called the PRogramme for Improving Mental health carE (PRIME) adopted and adapted the mhGAP in five LMICs, including Nepal, through which basic biomedical and psychosocial management of mental health problems was integrated into community-based health facilities [17]. At the community level, two of the strategies were developed to promote detection and raise awareness about mental health and the availability of treatment services: 1) an innovative case-finding tool called the Community Informant Detection Tool (CIDT) [18] and 2) a community sensitization programme. The CIDT is a simple paper-based form consisting of contextualized vignettes and associated pictures of mental health problems. Anyone with symptoms matching the prototype, impaired functioning level, or need for external support are referred to the health facility. The CIDT has been validated in the Nepali context [19] and has been effective in promoting health-seeking [20]. The community sensitization programmes, on the other hand, entail orientation on key concepts of mental health, myths and facts, types of mental disorders, and locally available services. Breuer et al. [21] noted that the case flow was higher at the health facility when community sensitization activities were conducted. In Nepal, both of these activities are conducted by lay community health workers called female community health volunteers (FCHVs) mobilized by the government of Nepal for the promotion of maternal and child health programmes [22, 23].

Although these strategies have been effective, they are limited to the general population with depression, alcohol use disorder, psychosis, and epilepsy. Baron [24] noted a lack of recording, reporting, and treatment of maternal depression in PRIME in Nepal. In Nepal, there is no nationally representative data on perinatal depression, but few studies conducted in postnatal depression in the last 5 years indicate that the prevalence varies between 15-34% [25–27]. In the Nepali context, where strict patriarchal norms are practiced, women are often dominated, subjugated, and are subject to violence [28]. Studies suggested that the rates of intimate partner violence against women was higher during pregnancy and postnatal period when they refused to have sex or gave birth to a female child [29]. Gender is a strong factor influencing causations, health-seeking behaviour, and experience of mental health problems, as well as its impact on sociocultural and economic aspects [30]. These gender-based differences necessitate a need for gender-sensitive and gender-specific mental health services [30–33].

Thus, the current study aims to adapt the existing community sensitization programme and CIDT to increase awareness and detection of maternal depression at the community level. The paper describes the adaptation process step by step.

## Methods

### Setting

Nepal is one of the poorest countries in South Asia and has a fragile health system. The country witnessed a massive earthquake in 2015, and the recent COVID pandemic has increased the risk of common mental health problems in the general population and more so among pregnant and postnatal women [34–36]. Maternal mental health care is limited; the country has only 0.05 psychiatrists per 100,000 [37], and antenatal care (ANC) and postnatal care (PNC) are limited to physical health.

This study was conducted in Chitwan, a district in the southern part of Nepal. General mental health services during the study period were provided from community health facilities through PRIME, and severe cases were referred to tertiary district hospitals [38]. However, no focused care was available for maternal mental health concerns.

### Data collection and analysis

In-depth interviews (IDI) were conducted by four female researchers with at least undergraduate degrees and work experience in mental health research. An additional two-day training was provided to increase familiarity with the study, its objective, and the study tools. The first author (PS), a female researcher for PRIME project with master’s degree and 5+ years’ work experience in public mental health research, conducted focused group discussion (FGD) accompanied by one note-taker. Data collection took place face-to-face in a private space at the health facility or at the community outreach clinic called *“gau-ghar”*. Each qualitative interview lasted between 30 minutes and one hour whilst the FGD lasted between 1 hour to 1 hour 30 minutes. The interviews were audio-recorded in Nepali, transcribed verbatim, and translated to English by a professional translator. Interviews were qualitatively analysed by the first author (PS) in NVivo 11 [39] using the framework approach [40]. The consolidated criteria for reporting qualitative research (COREQ) checklist was used to guide to report findings. (See Supplementary file 1)

### Study procedure

We conducted a four-step process to develop the content of perinatal depression and adapt the existing community sensitization program manual and CIDT.

#### Step 1: qualitative study

Pregnant and postnatal adult women (18 years and above) visiting ANC and PNC clinics or *Gau-Ghar* Clinic were screened by researchers for depression using the Edinburgh Postnatal Depression Scale (EPDS). The EPDS has already been validated in the Nepali context with a cut-off score of 13 [41]. Women exhibiting depressive symptoms were invited for IDIs. Semi-structured qualitative guide was used for IDI. Data saturation was assessed after completing 10 interviews (5 antenatal and 5 postnatal), and data collection concluded once no new information emerged. Additionally, five focus group discussions (FGDs) were conducted: two with health workers (*n*=13), two with FCHVs (*n*=16), and one with psychosocial counsellors (*n*=5) in their respective health facilities. Purposive sampling was used to select the FGD participants. All the FGD participants were previously trained on mental health through PRIME project and were engaged in mental health service delivery either at the community or health facility level. Researchers administered the informed consent form by reading through the information sheet, ensuring understanding, and answering any questions before participating in the IDIs and FGDs. Only those, who provided written consent, were engaged in the study.

Interviews and FGDs focused on local terminologies and expressions of perinatal depression, perceived causes, help-seeking behaviour, barriers to care, and possible intervention. The interviews with depressed women focused on their lived experiences, while the FGDs explored perinatal depression through the service providers’ and community’s perspectives. (See Supplementary file 2).

#### Step 2: draft preparation

The content of both CIDT and the existing community sensitization manual were modified and made specific to perinatal depression based on qualitative findings from Step 1. A list of symptoms exhibited by depressed antenatal and postnatal women and its perceived risk factors were prepared to adapt the CIDT. The content adaptation of the community sensitization manual was made taking into consideration the cultural nuances, metaphors, traditional worldviews and understandings outlined as important key issues for adaptation [42]. The draft was shared for review with the psychologist, who was also working for PRIME as a trainer and a supervisor to community psychosocial counsellors.

#### Step 3: workshop

A one-day workshop was conducted with the psychosocial counsellors (*n*=2) and community health workers (*n*=12). Five questions were asked to identify what changes needed to be made in the case vignettes: a) To what extent is the case vignette easy to understand? ; b) Has the case vignette included all the major symptoms? ; c) What changes need to be made in the case vignette? ; d) How can the case vignette be made simpler? ; and e) Do you think any information should be added? The workshop participants also reviewed and revised the community sensitization manual content during the workshop.

#### Step 4: finalization

For the final review, the revised draft was shared with a psychiatrist working at Transcultural Psychosocial Organization Nepal, a local nongovernment organization implementing PRIME in Nepal. The psychiatrist had extensive experience conducting training and supervising community-based health workers, as well as adapting CIDT to the post earthquake context.

## Results

Altogether, 293 perinatal women visiting ANC, PNC, and *"gau-ghar"* clinics were screened using the EPDS, out of whom 36 (12.3%) had depressive symptoms. Six women refused to be interviewed, 3 did not complete the interview, and one did not report depressive symptoms in the follow-up meeting. More than half (54%) of the women engaged in the interview were in the postnatal period, and most women (77%) were between 18-25 years with a mean age of 24.6 years. Four-fifth of the participants were not engaged in any income generating activity (80.8%) but only one-third reported that the family’s income was insufficient to meet their needs (34.6%). Further breakdown of participants by religion, caste, education, occupation, income, and number of pregnancies are presented in Table 1. A significant number of the FGD participants were female (88%) and had attended some level of formal education (97%). Other sociodemographic details are presented in Table 2.

**Table 1:** Socio-demographic information of the depressed women

	**Total antenatal women (*n*=12)**	**Total postnatal women (*n*=14)**	**Total participants (*N*=26)**
**Age**			
18-25	9 (34.6%)	11 (42.3%)	20 (76.9%)
26-30	2 (7.7%)	0	2 (7.7%)
31-35	1 (3.8%)	2 (7.7%)	3 (11.5%)
36-40	0	1 (3.8%)	1 (3.8%)
**Caste/Ethnicity**			
Brahmin/Chhetri	4 (15.4%)	4 (15.4%)	8 (30.8%)
Dalits	6 (23.1%)	7 (26.9%)	13 (50%)
Chaudhary/Tharu	1 (3.8%)	2 (7.7%)	3 (11.5%)
Janajati	1 (3.8%)	1 (3.8%)	2 (7.7%)
**Religion**			
Hindu	11 (42.3%)	9 (34.6%)	20 (76.9%)
Christian	0	4 (15.4%)	4 (15.4%)
Others	1 (3.8%)	1 (3.8%)	2 (7.7%)
**Education**			
No formal education	1 (3.8%)	3 (11.5%)	4 (15.4%)
Primary level (1-5)	3 (11.5%)	3 (11.5%)	6 (23.1%)
Lower secondary (6-8)	4 (15.4%)	2 (7.7%)	6 (23.1%)
Higher Secondary (9-12)	4 (15.4%)	4 (15.4%)	8 (30.8%)
Undergraduate	0 (0%)	2 (7.7%)	2 (7.7%)
**Occupation**			
Agriculture	9 (34.6%)	12 (46.2%)	21 (80.8%)
Wage/seasonal labourer	1 (3.8%)	2 (7.7%)	3 (11.5%)
Business	2 (7.7%)	0 (0%)	2 (7.7%)
**Income Sufficiency**			
Enough throughout the year	7 (26.9%)	10 (38.5%)	17 (65.4%)
A little insufficient	1 (3.8%)	0 (0%)	1 (3.8%)
Not sufficient	4 (15.4%)	4 (15.4%)	8 (30.8%)
**Pregnancy**			
Primagravida/First pregnancy	7 (26.9%)	6 (23.1%)	13 (50%)
Multigravida/Multiple pregnancy	5 (19.2%)	8 (30.8%)	13 (50%)

**Table 2:** Socio-demographic information of the service providers

**Respondent Type**	**Health Worker (*n*=13)**	**Psychosocial Counsellors (*n*=5)**	**FCHVs (*n*=16)**	**Total (*N*=34)**
**Sex**				
Male	2 (5.88 %)	2 (5.88 %)	0 (0%)	4 (11.76%)
Female	11 (32.35%)	3 (8.82%)	16 (47.05%)	30 (88.23%)
**Age**				
25-35	4 (11.76%)	2 (5.88 %)	2 (5.88 %)	8 (23.52%)
36-45	4 (11.76%)	2 (5.88 %)	8 (23.52%)	14 (41.17%)
46 and above	5 (14.70%)	1 (2.94%)	6 (17.64%)	12 (35.29%)
**Education**				
No formal education	0 (0%)	0 (0%)	1 (2.94%)	1 (2.94%)
Primary	0 (0%)	0 (0%)	1 (2.94%)	1 (2.94%)
Lower Secondary	0 (0%)	0 (0%)	11 (32.35%)	11 (32.35%)
Higher Secondary	11 (32.35%)	1 (2.94%)	3 (8.82%)	15 (44.11%)
Undergraduate	2 (5.88 %)	4 (11.76%)	0 (0%)	6 (17.64%)
Graduate	0 (0%)	0 (0%)	0 (0%)	0 (0%)

### Step 1: qualitative study

#### Common depressive symptoms and local terminologies

The study participants used emotional and psychological expressions such as sadness, loss of interest, and feelings of worthlessness locally expressed as *“naramailo/dukha lagne”, “alchi huney”, and “bacheko bekkar lageko”* to express their depressive symptoms. Few symptoms of anxiety, such as rumination, extreme worries, and restlessness, were frequently mentioned. Too much *“tension”* was linked with loss of concentration and forgetfulness expressed as though their *“minds have stopped working”* or made them go *“completely blank”*. Antenatal women complained about having difficulty in the body that hindered their capacity to conduct daily activities, which they linked with frustration expressed as *“birakta lagne”* and* “dikka lagne”*. Postnatal women associated having a baby as being caught in a web of hassles *“jhanjhat ma faseko jasto hune”* and were frustrated about their changed lifestyle marked by disturbed sleep and lack of time for self-care. Unable to meet demands from the family and having to take care of the baby made them stressed, guilty, and sometimes angry and irritated. Few reported having suicidal thoughts. A postnatal woman shared that she would grow more furious at her children at times when she was deeply troubled.

“It’s like you know, this baby makes me feel as if I am caught up in problems something like that, which makes me feel very irritated “jhingaleko”. I don’t feel like taking care of this baby very well. I wish some other person would look after this baby. I don’t have energy within me. I am growing lazy.” – IDI with Postnatal Woman

The FGD participants cited behavioural symptoms such as isolating from others, getting irritated or angry easily, staring at blank spaces for a long time *“tolaune”*, looking depressed *“jhokrayera basne”*, and being single-mindedness 1 “*ekohoro huney”* as most common. Other symptoms, such as reduced interest and impaired relationships with their infants and family members, were also noted. (See Table 3)

**Table 3:** List of symptoms from qualitative study

**S.N.**	**Symptoms**	**Cultural Expression**	**Frequency**	**Source**
1.	Worries especially about future	*“piir lagne” “dukha lagne” “tension”*	20	Interviews with antenatal and postnatal women
2.	Preferring to stay alone; stay far from home; don’t feel like seeing anyone else	*“eklai basna man lagcha”, “tadha gayera basnu maan lagne”, “ghar chodera hidna maan lagne”, “kosailai herna pani maan lagdaina”*	17	Interviews with antenatal and postnatal women
3.	Fatigue, loss of energy or loss of interest in work (expressed in terms of laziness or weak body)	*“alchii lagne”, “alasyata”, “sarir bhari huney”*	14	Interviews with antenatal and postnatal women
4.	Irritation (feel irritated to talk to anyone; feeling like nobody would come and to talk to her)	*“jhijo lagne”; “jhingaleko”, “koi pani ma sanga nabolidiye hunthyo jasto lagne”;“Aru le bolda jharko manne; koi herna maan nalagne”*	13	Interviews with antenatal and postnatal women
5.	Thoughts about suicide or self-harm or thinking that it would be better off to die	*“marnu maan lagne” “marau marau lagne” “bachnu bhanda ta marekai thik” “marey dhukkai hunthye” “afi lai hani garne soch”*	11	Interviews with antenatal and postnatal women
6.	Stare at a blank space	*“tolaune” “tolayera basne”*	9	FGDs with counsellors and health workers and few women
7.	Restlessness	*“kati khera kata jaam huney”, “chaatpaati huney”.*	9	Interviews with antenatal and postnatal women
8.	Sleeplessness (mainly due to piir-worries/rumination/stress)	*Nindra nalagne (translation)*	8	Interviews with antenatal and postnatal women
9.	Loss of appetite	*khana maan lagthena*	8	Interviews with antenatal and postnatal women
10.	Depressed/ frustrated face	*“jhokrayera basne”, “udaas dekhincha” “uraath biraath dekhiney”*	7	FGDs with counsellors and health workers and few women
11.	Ruminating/contemplating	*“maan ma dherai kura khelne”, sochdai basirahaney*	7	Interviews with antenatal and postnatal women
12.	Angry or furious even in trivial matters or without reason	*“chin-chin mai ris uthne”; jolai dekhey ni ris uthyo*	7	Interviews with antenatal and postnatal women
13.	Anxious (something might go wrong or not being able to take care of the baby)	*“chinta lagne” “aatiney”*	7	Interviews with antenatal and postnatal women
14.	Forgetfulness	*Birsiney*	6	Interviews with antenatal and postnatal women
15.	Crying	*“runu maan lagne”*	6	Interviews with antenatal and postnatal women
16.	Physical complaints like headache, stomachache	“tauko dukhne”, “pet dukhne”	5	Interviews with antenatal, FGDs with health workers and psychosocial counsellors
17.	Sad/Unhappy (esp not receiving support from family); feeling bad	“naramailo lagne”, “namajja lagne”	4	Interviews with antenatal and postnatal women
18.	Feeling worthless, useless, hopeless	“bacheko bekkar lageko”	4	Interviews with antenatal and postnatal women
19.	Lack of self-care		4	Interview with postnatal woman, FGDs with health workers
20.	Looks worried	“niraas”/“chintit”	4	Interviews with postnatal woman; FGDs with health workers and psychosocial counsellors.
21.	Dark face	“adhyaro mukh”	4	Interviews with postnatal woman; FGDs with health workers and psychosocial counsellors.
22.	Lack of concentration	"dhyan kata kata huney”	4	FGDs with health workers and psychosocial counsellors.
23.	Being single-minded	“ekohoro huney”	3	FGDs with health workers and psychosocial counsellors.
24.	Frustration	“birakta lagne”, “dikka lagne”, “baccha bhako dekhera dikka lagne”	3	Interviews with antenatal and postnatal women
25.	Lack of zeal (explained as effortless and unhappy talking to others)	“maan naramayera boleko, naramailo tarika le boleko”	3	Interviews with postnatal woman; FGDs with health workers and psychosocial counsellors.
26.	Guilty; self-blame	“doshi thanney”	3	Interviews with postnatal women
27.	Nightmares (fear of delivery)	naramro sapana	2	Interviews with antenatal women
28.	Apathetic	Aruko wasta nagarney	2	FGDs with health workers and psychosocial counsellors.
29.	Not able to control the mind	Dimag fuskinu ateko jasto huney	2	Interviews with postnatal women
30.	Feel heavy hearted	Maan bhari huney	1	Interviews with antenatal women
31.	Caught up in trouble	“jhanjhat ma faseko jasto hune”	1	Interviews with postnatal women
32.	Pounding heart	Maan bhut bhut huney	1	FGDs with psychosocial counsellors
33.	Take alcohol, cry and shout, or mumble to self	dherai pir parera rakshi khancha, binakaran karaucha, runcha wah afai sanga bolirakcha	1	FGDs with psychosocial counsellors
34.	Burning sensation	Maan bhat bhat polney	1	FGDs with psychosocial counsellors
35.	Difficulty breathing	Saas fernu garho huney	1	FGDs with psychosocial counsellors
36.	Feeling like something is blocking the heart	Mutu ma k adkeko jasto huney	1	FGDs with psychosocial counsellors

#### Perceived causes

Lack of support, financial constraints, household work burden, unplanned pregnancy, cultural preference for a son, and painful experience in the past were commonly identified risk factors for perinatal depression. After marriage, many women shared having difficulty adapting to a different culture, having limited freedom, and lacking decision-making power at their husband’s home. Husbands were portrayed as a major pillar of strength but lacking their and family’s support, and having unsatisfying marital relationships caused them to feel lonely, sad, and hopeless.

#### Understanding of the problem

Only two women with a previous history of depression had sought care for depressive symptoms from the hospital. Conventional thoughts and religious beliefs that mental health problems were the outcomes of sinful acts in the past or failure to follow the right religious practice properly were common among the respondents. When the gods/lineage gods are angry *“devi deuta risako”* or unhappy with the present generation’s ignorance about pleasing god, then it is was believed that they would afflict the ones that stray away from the traditional religious practices. Still several others blamed fate *“sapta bigreko”* or luck *“graha dasha lageko”*. When people displayed unusual behaviours, they were perceived to have been possessed by the spirits hence seek care from the traditional and faith healers.

“People believe that it happened because the gods are angry *“devi deuta risako”* or ancestral gods are angry *“kul risako”* when their problems grow severe. Medication takes time to show the effect; hence, they think that it would be treated by traditional healers. We still have such culture and beliefs.” - FGD with health worker

Poor awareness about service availability, service types, and beliefs that taking medication during pregnancy is harmful had barred women from seeking care from the health facility.

“[...] I didn’t know that I should go to hospital when I have tension or worries. I thought that one goes to hospital only when s/he is sick. I didn’t know that.” – IDI with postnatal woman

Insecurities of being mocked or labelled with stigmatizing names such as having a loose mind *“dimag fuskeko”*, mad *“baulaha”*, loser *“kehi garnu nasakne”*, crazy *“pagal”*, and *“psycho”* made some of them reluctant to seek support.

#### Possible intervention for perinatal depression

Citing lack of awareness and low detection of perinatal depression in the community, the FGD participants shared that the key to tackling the problem is through early detection. They emphasized educating husbands and family members. The health workers particularly underscored the importance of information dissemination through printed materials (brochures, leaflets, pamphlets), mass media, community sensitization programmes, and integrating the information about mental wellbeing in the school curriculum.

The depressed women thought that women with similar experience can empathize and thus be able to identify such problems in others. A safe peer support group, if established, would let them share their problems and support one another. They also stressed educating the head of the family or the key community persons:

“[...] if the head of the family is taught or transferred knowledge about it, then it would be better. I find that a better option because if the head of the family says something, everyone believes him. If you educate me about this and if I tell about these things in my family, I think nobody would take me seriously.” - IDI with postnatal woman

### Step 2: draft preparation

A table containing the list of symptoms, their cultural expression, frequency, and source was prepared. Initially, 47 symptoms were listed from the qualitative data. Similar symptoms were combined. The final list contained 36 symptoms (see Table 3). These symptoms were ranked based on the frequency of their use by the study participants. Under sources, it was indicated whether the symptom was commonly reported by antenatal or postnatal women or the service providers. Two case vignettes were prepared containing 13-14 frequently used symptoms for antenatal and postnatal depression. Frequently endorsed risk factors were used to create a context in the case vignette. The draft case vignettes were shared with the psychologist for feedback. The psychologist suggested including symptoms related to four areas: physical, emotional, thoughts, and behaviour. The draft was reviewed to ensure that all these symptoms were incorporated, especially in the postnatal vignette where physical symptoms were missing.

Following the content outline of four mental health problems in the community sensitization manual, a subsection on perinatal depression was created under depression, where case vignettes of the adapted CIDT for antenatal and postnatal depression, general information about perinatal depression, its causes and symptoms from the qualitative study were incorporated (see Table 4 for the outline). Since most of the causes and symptoms were similar to general depression, only unique features (e.g., cultural preference for a son, lack of husband//family support, and impaired relationship with husband) were listed in the perinatal depression section.

**Table 4:** Adaptation of community sensitization manual

Adapted Version
(The titles in **Bold** indicate areas where changes have been made; the ***Bold, and Italics*** text briefly describes the changes)
1. Introduction
• Background
• Introduction to the manual
• Content of the manual
• Process of community sensitization programme
2. Psychosocial Concept (30 minutes)
• What is psychosocial?
• Psychosocial wellbeing and problems
• Causes of psychosocial problems
• **Symptoms of psychosocial problems *(symptoms added)***
• How to identify psychosocial problems
• **Cultural expressions of psychosocial problems *(cultural expressions added)***
• Evaluative question: What do you understand by psychosocial?
**3. Mental Health Concept *(changed from 1.5 hours to 2 hours)***
• Mental Health
• Mental health problems
• Causes of mental health problems
• Symptoms of mental health problems
• **Myths and facts about mental health problems *(few myths and facts about mental health and perinatal depression added)***
• Types of mental health problems
• Depression
• Case Vignette (from CIDT)
• **Introduction to depression *(Definition revised in the workshop)***
• **Causes of depression *(Few common causes from the workshop added)***
• **Symptoms of depression *(Few common symptoms from the workshop added)***
• **Perinatal depression *(This sub section was added)***
• **Case Vignette of antenatal and postnatal depression *(from CIDT)***
• **Introduction to perinatal depression *(Definition derived from the workshop)***
• **Causes of perinatal depression *(Common causal factors were added as a result of the qualitative study and the workshop)***
• **Symptoms of perinatal depression *(Common symptoms were added as a result of the qualitative study and the workshop)***
• Alcohol Use Disorder
• Case Vignette (from CIDT)
• Introduction to alcohol use disorder
• How to identify people with alcohol use disorder?
• Causes of alcohol use disorder
• Symptoms of alcohol use disorder
• Epilepsy
• Case Vignette (from CIDT)
• Introduction to epilepsy
• Causes of epilepsy
• Symptoms of epilepsy
• Psychosis
• Case Vignette (from CIDT)
• Introduction to psychosis
• Causes of psychosis
• Symptoms of psychosis
4. Stigma (10 minutes)
• Impact of stigma on wellbeing
• **How to tackle stigma? *(includes some practical strategies that can help)***
5. Treatment (20 minutes)
• **Role of family to help people with mental health problems *(Findings from the literature review and workshop added)***
• Available psychosocial and mental health services at the health facilities
6. **References *(Additional references)***

### Step 3: workshop

Participants were divided into three groups of 4-5 participants each. A task to define perinatal depression, its causes, and symptoms in a simple language was assigned to each group, which was reviewed and finalized in the large group. Factors such as unplanned pregnancy, forced pregnancy, stillbirth, short birth spacing, and early or late pregnancy unique to perinatal depression were added. Although symptoms related to fatigue and impact on daily functioning were also mentioned in the depression component, they were also added to the perinatal depression section. “Fatigue” in perinatal depression was explained more in terms of laziness caused by physiological difficulties, while “impact on daily functioning” was more related to difficulty carrying out household chores.

Common misconceptions relating to depression, such as it being caused by spirit possession, angry gods, or ill fate, were listed under the “myths” section followed by “facts”. The large group indicated the importance of the family's role and preparation for the baby’s arrival. The “Thinking Healthy Programme” [43], an intervention developed by the WHO for perinatal depression, was reviewed, and the "role of family" section was added to the manual.

The workshop participants were then shown the antenatal and postnatal depression case vignettes for CIDT prepared in Step 2. Both the antenatal and postnatal case vignettes were reported to be simple, clear, and easy to understand. Given the limitation that these case vignettes should be brief, the participants indicated that the case vignettes had included all the major and common symptoms and thus needed no changes.

### Step 4: finalization of the draft

**Fig. 1:**
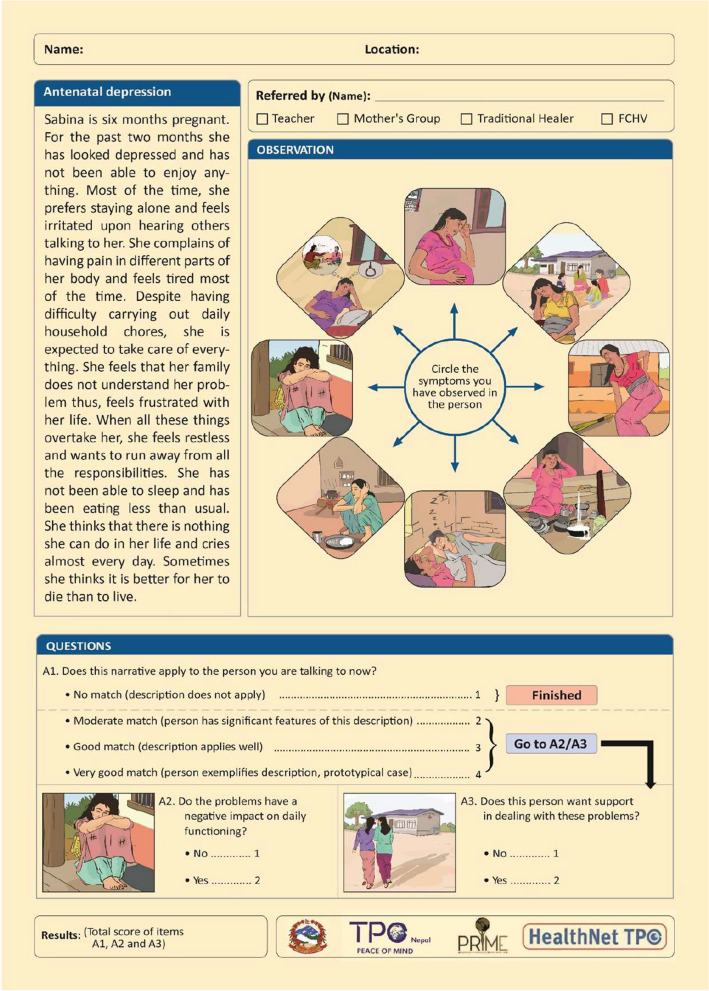
Final CIDT for antenatal depression

**Fig. 2:**
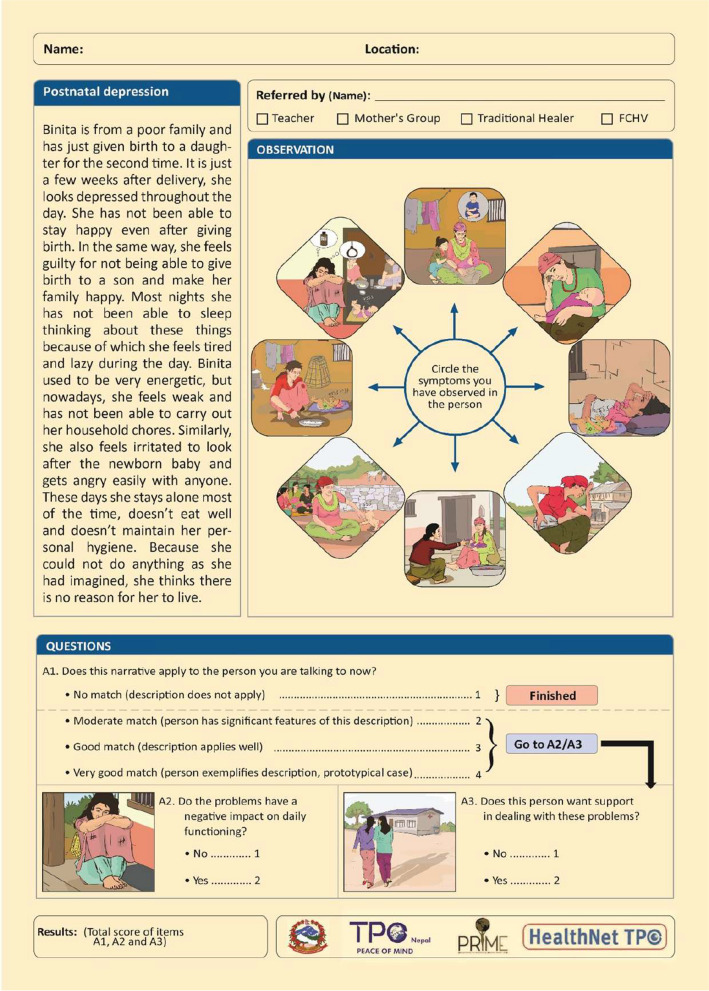
Final CIDT for postnatal depression

For the finalization of CIDT, the case vignettes were sent to a psychiatrist for review and feedback to ensure that major symptoms were correctly presented in the case vignette. Although the case vignettes were found appropriate, the psychiatrist suggested that three common symptoms of depression related to low mood, fatigue, and decreased interest should be prioritized and should be mentioned before any other symptoms. Therefore, for the antenatal case vignette, symptoms related to low mood expressed by depressive feelings and loss of enjoyment were mentioned first, followed by behavioural changes and physical changes. Depressed mood, self-blame, worries, and hopelessness were added in order in the postnatal vignette. Additionally, sleeping disturbance and loss of appetite, which are common complaints by depressed patients, and the duration of the persistence of symptoms were added in both vignettes. Furthermore, "self-blame" was replaced with feelings of guilt and "not eating" with diminished appetite. In the postnatal case vignette, a sentence about the protagonist’s worries about rearing up the children was removed as per the psychiatrist’s recommendation since it relates more to anxiety and not depression alone. After the revision, a consultant artist was hired to develop pictures to include in the CIDT (see Figs. 1 and 2).

## Discussion

We followed a four-step qualitative process to adapt the CIDT and community sensitization manual for perinatal depression. The first step entailed understanding the cultural expression of depression, its risk factors, and possible interventions through a qualitative study with perinatal women identified with depression, health workers, and psychosocial counsellors. Perinatal women, who exhibited severe symptoms in the screening tool or reported having suicidal thoughts during the qualitative interview, were followed up by a psychosocial counsellor for free counselling services. Findings from the qualitative study were then used to develop a draft in the second step. Third, the draft was refined in a workshop with the community health workers and was reviewed by the psychologist. Finally, a few modifications were made as per the psychiatrist's recommendation, and the tool and manual were finalized.

The maternal and child health is one of the priority programmes of the government of Nepal, however, it is exclusively focused on physical health [44]. In a country where suicide is the leading cause of death in women of reproductive age [45], the target set by the government to achieve maternal and infant wellbeing may be jeopardized if mental health is neglected. Thus, strategies to promote detection and health-seeking both at the health facility and community level are needed for the timely management of maternal depression [10, 46].

Detection can be improved by using screening tools, structured diagnostic assessments, or increasing awareness. Screening tools are often the first step in the management of perinatal mental health problems [47–49] and have long been used for case finding [50]. However, given its limitations of being time consuming when administered to illiterate patients [51], a greater risk of misinterpretation and misunderstanding with self-administered tools, and a lack of cultural validity [46, 51, 52] necessitate culturally sensitive detection tools. The strength of this study is that it has utilized a community participatory approach to adapt the CIDT and community sensitization programme. In a context where mental health problems are highly stigmatized, using local idioms can be more acceptable and advantageous to improve help-seeking behavior [53]. The CIDT uses culturally appropriate, non stigmatizing case vignettes to describe mental illness that are used by community gatekeepers to identify potential cases and bridge them to treatment [18]. The tool has been developed, validated, and found effective in three countries: Nepal [20], Pakistan [54, 55], and South Africa [56].

Additionally, it is imperative to address misconceptions and disseminate information about the problem and services to promote health-seeking [57]. Conventional thoughts and religious interpretations that mental health problems are caused by sinful acts, angry gods or bad luck are still common [58]. The understanding of emotional problems as personal problems deterred our participants from seeking help from the health facility. Furthermore, the health facility was portrayed to be limited to pharmacological services and for the treatment of physical problems only. Similar perceptions prevailed among Bangladeshi women, where health workers were perceived to be helpful for physical problems and an “inappropriate” person to talk to about their emotional problems [59]. The fear of being prescribed medicine was another reason deterring participants from sharing problems with health workers. Previous studies suggest that perinatal women are especially reluctant to use medicine, fearing that it would affect their foetus/infant [60].

Furthermore, low education, less decision-making capacity, distance to the health facility, transportation problems, and financial problems have been frequently cited for the underutilization of maternal health services in Nepal [25, 61, 62]. A study in Afghanistan, where family and cultural factors, posed barriers to treatment adherence, had recommended awareness programmes to tackle stigma, and promote health-seeking [46]. Mass education through awareness programmes is a widely used strategy in public health to address misconceptions and heighten awareness [63–65]. Recently, the use of media and mobile applications for information sharing has increased. Although these can be cost effective and have larger coverage, face-to-face events and physical gatherings in close-knit communities can have profound effects in the rural context of Nepal. Coupled with CIDT, which enables community volunteers to trace potential mental health cases at the community level, these community strategies can be crucial to promote identification and health-seeking for mental health problems. A recent study that adapted CIDT in the South African context also highlighted the need for a psychoeducation component to address misconceptions and relay correct information about mental health problems [56].

The study also has several implications for future research and implementation into designing public mental health interventions. First, experiences and expressions of mental health varies in different contexts [66]. Hence, it is imperative for mental health interventions to be attuned to local culture. Future research can adopt participatory approaches to focus on understanding the ethnopsychology of mental health problems while tailoring psychological interventions to local contexts. Second, multidisciplinary approaches to mental health treatment may be required. Consistent to a previous study [67], our study participants also highlighted the practice of consulting traditional healers. Ethnographic studies and approaches to cultural psychiatry may give future directions to integrate cultural practices alongside biomedical approaches to address mental health concerns. Third, it is important to identify the target audience when developing these community interventions. For example, one of our participants shared that educating the woman may not be sufficient since their opinions are not valued. Women, especially daughters in law, hold the lowest position where their decision is made by others [33]. It is therefore crucial to engage their family members. Studies have found that women whose husbands were literate were more supportive and had higher service utilization rates [68]. The WHO’s recommended Thinking Healthy Programme, a community-based intervention for perinatal depression, also encourages and includes components for family engagement [43]. Apart from family members, educating key community people who are revered in the community can also be beneficial, which is in-line with key recommendations highlighted by Tomlinson et al. [46]. Lastly, future research is also needed to evaluate the effectiveness of the developed CIDT and community sensitization programme in detecting and promoting help seeking behaviour of women with perinatal depression.

### Limitations

Nepal is a diverse country with varied ethnic groups and cultures. This study was conducted in the Chitwan district of the Terai region and may not necessarily be representative of the hilly and mountainous region. It may only be relevant to communities with similar ethnic and socioeconomic characteristics. Second, perinatal anxiety is common and often comorbid with depression. This tool is only limited to depressive symptoms and may not necessarily be beneficial to identify common mental health problems. Third, engagement of sociologists or medical anthropologists could have yielded valuable insights in the adaptation process. However, we were not able to include them in the workshop or in the finalization process. This limitation underscores the need for interdisciplinary collaboration to enhance the cultural sensitivity and relevance of interventions in future research endeavours.

## Conclusion

In a context where cultural and community factors impede health-seeking, health services should be available both in the health facility and beyond [46]. Focused community strategies for perinatal mental health can have profound effects on timely identification and promotion of health-seeking. However, they must be complemented by availability of health services. This study was conducted in community health facilities where basic mental health services were available. Rather than separating mental health from physical health, mental health component should be embedded in existing maternal health programmes [15]. The FCHVs in Nepal provide door-to-door service to pregnant and postnatal women and conduct monthly mother group meetings that can serve as the best opportunity to use CIDT and the community sensitization manual to identify potential women with perinatal depression, create awareness and provide psychoeducation. Simultaneously, capacity building of midwives, who are often the first point of contact for pregnant and postnatal women in community-based health facilities in Nepal, is crucial. Furthermore, studies are required to validate and evaluate the effectiveness of community awareness programmes and CIDT for timely identification and effectiveness in promoting health-seeking for maternal mental health. More studies are required on what components of mental health should be integrated into the antenatal and postnatal care package.

### Supplementary Information

**Supplementary Material 1.**

**Supplementary Material 2.**

## Data Availability

Authors interested in the study datasets should submit requests via the PRIME consortium Expression of Interest form available online through https://cpmh.org.za/primesite/contact-prime.html ..

## Abbreviations

ANC: Antenatal care
CIDT: Community informant detection tool
EPDS: Edinburgh Postnatal Depression Scale
FCHV: Female community health volunteers
IDI: In-depth Interview
FGD: Focus Group Discussion
LMIC: Low- and middle-income countries
mhGAP: Mental health Gap Action Programme
PNC: Postnatal Care
PRIME: Programme for Improving Mental Health Care
THP: Thinking Healthy Programme
WHO: World Health Organization

## Footnotes

1 ‘Single-mindedness’ is a local phrase used to explain rumination or doing the same thing persistently for a prolonged period of time without considering anything else
