# Supplementary material for: A qualitative study on the adaptation of community programmes for the promotion of early detection and health-seeking of perinatal depression in Nepal
Source: BMC Womens Health. 2024 May 4;24:273. doi: 10.1186/s12905-024-03122-y (PMC11069154; doi:10.1186/s12905-024-03122-y)
Supplement: Supplementary file 2 — Supplementary Material 2. [file 12905_2024_3122_MOESM2_ESM.docx]

# Interview Schedule for Perinatal Women with Depressive Symptoms

| **S.No.** | **Theme** | **Core Questions** | **Probe** |
| --- | --- | --- | --- |
| 1. | **Ice Breaker** | Can you tell me about your visit *[today or refer to the date EPDS was administered]*? | - Which ANC/PNC visit is/wasit? - Problems - Type of service sought/received |
| 2. | **Pregnancy**  **(For Antenatal Women)** | What were your feelings when you found out that you were pregnant? | - Things that made happy and sad after finding out about pregnancy - Feelings related to those happy and sad moments - Spoken to anyone (family, friends) about your pregnancy - Expectations from friends and family during pregnancy |
|  | **Delivery**  **(For Postnatal women)** | Now that you have already given birth to a baby, can you tell me about your feelings? | - Gender of the baby - Most enjoyable and bothersome experience being a mother - Impact on daily life/schedule - Support from immediate family members and family - Expectations from immediate family members and family |
| 3. | **EPDS** | Please tell me more about the problems (*repeat the symptoms the respondent has listed in the EPDS scale*) you are currently experiencing. | - Onset (each problem) - Perceived causes (psychological, physical and social) - Impact on daily life - Spoken to anyone (family, friends) about your pregnancy - Community's understanding about the problem (common symptoms/terms/*"idioms of stress"* used to refer people with such problems) |
|  |  | Please share me your experience of a day when these feelings are really bad? | - Feelings at such times - Coping mechanism |
| 5. | **Help Seeking and Pathways to Care** | What do the community people do when they have these problems?  How are problems like these treated at the community? | - Available services (Traditional treatment, modern treatment) |
|  |  | Personal level: Could you please explain me in detail what have done when you had such problems? | - Consulted sources for the problem - Pathways to care - Reasons to seek care from certain source - Advantages and disadvantages of each source - Plan to continue from the same source/ recommend others to seek service from that source. |
| 4. | **Detection of perinatal depression** | There may be others who may have been undergoing similar problems as yours. Do you think their problems are identified and treated properly? | - How and who could identify - Potential advantages and disadvantages - Protective factors |

# Focus Group Discussion Guide for Counsellors, Health workers and FCHVs

| **S.No.** | **Themes** | **Core Questions** | **Probe** |
| --- | --- | --- | --- |
| 1. | **Icebreaker Question** | **For Counsellors & FCHVs:** Can you tell us about mental health situation in the district? | - Common mental illnesses - Common practice to deal with mental illness |
|  |  | **For Health Workers:** Can you tell us about your health facility? | - General health services - Mental health facilities |
| 2. | **Working experience in mental health** | **Care (For Counsellors & Health workers):** What are the general components of care you have been providing for people with mental disorders?  **For FCHVs**: What is your understanding about depression and perinatal depression?  How are you involved in mental health programme in your community? |  |
|  |  | **Identification:** How do you identify someone having depression? | - Any instruments, standards, guidelines - Common expressions/ symptoms/ idioms of stress used by people with depression |
|  |  | **Vulnerability:** Who do you think are more at risk for depression? (gender, caste/ethnicity/poor)  Why particular <group that the participants mention> are more at risk? |  |
| 3. | **Local beliefs and attitudes towards depression** | How does the community perceive someone with   - Depression? - Perinatal Depression? | - Local understanding about depression & perinatal depression (Any association with witchcraft or any superstitious beliefs or cultural beliefs) - Perceived causes - Common terms/metaphors used to refer depression/perinatal depression - Behaviour towards people with depression (Stigmatizing behaviour) |
| 4. | **Help seeking and pathways to care for depression** | What is done when people have   - Depression? - Perinatal Depression? | - Available services at the community (traditional and modern care) - Common practices - Pathways to care - Facilitators and barriers |
| 5. | **Detection of perinatal depression** | **Community:** Do you think perinatal depression is prevalent in your community?  How are they identified at the community level? | - Who could identify? - How could they be identified? - Barriers to identify perinatal depression |
|  |  | **PHC (for health workers only):** How are perinatal cases detected at the health facility? |  |
